# Xiaoyaosan, a traditional Chinese medicine, inhibits the chronic restraint stress-induced liver metastasis of colon cancer *in vivo*

**DOI:** 10.1080/13880209.2020.1839513

**Published:** 2020-11-05

**Authors:** Lu Zhao, Xiaodan Zhu, Yiqun Ni, Jianliang You, Ao Li

**Affiliations:** a Department of Oncology, Wuxi Traditional Chinese Medicine Hospital, Wuxi, China; b Department of Traditional Chinese Medicine, Yongchuan Hospital of Chongqing Medical University, Chongqing, China

**Keywords:** Colorectal cancer, tumour metastasis, psychological stress

## Abstract

**Context:**

Xiaoyaosan (XYS), a traditional Chinese medicine (TCM), has been widely used to relieve a variety of disorders caused by depression.

**Objective:**

This study evaluates the effect of XYS against tumour metastasis in a chronic restraint stress mouse model.

**Methods and materials:**

Forty C57BL/6J mice were randomly divided into four groups, including blank-control (BC), blank-stress (BS), XYS-control (XC) and XYS-stress (XS). BS and XS groups were exposed to immobilization stress for 2 h per day for 28 days commencing seven days before tumour cell injection. XC and XS groups were given a gavage of XYS (1516.67 mg/kg) before chronic immobilization stress. Mice were injected with HT-29 colon cancer cells in the spleen to produce liver metastasis. After 28 days of injecting with HT-29 cells, flow cytometry, western blot, PCR and immunohistochemical staining were performed to uncover the role of chronic restraint stress and XYS in the liver metastasis of colon cancer.

**Results:**

Metastatic liver weight of mice in XS group (3.33 ± 0.18 g) was significantly lower than BS group (4.01 ± 0.27 g). Chronic restraint stress significantly increased CD11b^+^F4/80^+^ and CD11b^+^Gr^lo^Ly6C^hi^ cell infiltration. XYS treatment significantly decreased the CD11b^+^F4/80^+^ tumour-associated macrophage (TAM) population and CD11b^+^Gr^lo^Ly6C^hi^ myeloid-derived suppressor cell (MDSC). TGF-β, IL-6, MMP-9 and VEGF in spleen tumours significantly decreased in XYS group. XYS also reduced VEGF and CD31 in hepatic metastatic tissue, which were elevated by chronic restraint stress.

**Conclusions:**

XYS may successfully inhibit chronic-stress-induced liver metastasis. Results suggest that XYS may have therapeutic value for colorectal cancer treatment.

## Introduction

Colorectal cancer (CRC) is the third leading cause of cancer death worldwide, and liver metastasis is the major cause of CRC mortality (Siegel et al. 2014). Complete metastases resection with negative histological margins while preserving sufficient hepatic parenchyma is the only treatment that has been shown to significantly improve the long-term survival rate of patients with CRC liver metastasis (CRLM). However, the majority of patients are not eligible for surgical resection at the time of diagnosis (Ito et al. 2010; Wang et al. 2014).

Psychological stress can evoke negative effects on the neuroendocrine system, brain function and sympathetic nervous system. Multiple lines of evidence have indicated that chronic stress promotes the initiation and progression of cancer (Le et al. 2016; Nilsson et al. 2017; Joshi et al. 2018). Adrenaline and noradrenaline are the most important catecholamine hormones released during stress that may influence cancer progression (Thaker et al. 2006; Eng et al. 2014; Krizanova et al. 2016).

As a safe traditional Chinese medicine (TCM) preparation used clinically, Xiaoyaosan (XYS) has been widely used in China to relieve a variety of disorders caused by depression (Zhang et al. 2012). XYS was first reported in *Taiping Huiming Heji Jufang* (960–1279 AD) and is comprised of eight TCMs as shown in Table 1. The functions of XYS include soothing the liver, invigorating the spleen, and nourishing the blood. A previous study has reported that XYS may induce apoptosis and inhibit 4T1-induced tumour growth (Chen et al. 2012).

**Table 1.** The contents of each crude medicinal component in 10 g granulated XYS.

Herbal name	Chinese name	Latin binomial	Family	Amount
Chinese Thorowax Root	Chaihu	*Bupleurum chinense* DC.	Umbelliferae	8.1 g
Radix Angelicae Sinensis	Danggui	*Angelica* sinensis (Oliv.) Diels.	Umbelliferae	8.1 g
Radix Paeoniae Alba	Shaoyao	*Paeonia lactiflora* Pall.	Paeoniaceae	8.1 g
Rhizoma Atractylodis Macrocephalae	Baizhu	*Atractylodes macrocephala* Koidz	Compositae	8.1 g
Poria	Fuling	*Poria cocos* Wolf.	Polyporaceae	8.1 g
Radix Glycyrrhizae	Gancao	*Glycyrrhiza uralensis* Fisch	Leguminosae	8.1 g
Herba Menthae	Bohe	*Mentha haplocalyx* Briq.	Lamiaceae	3.9 g
Rhizoma Zingiberis Recens	Shengjiang	*Zingiber officinale* Rosc.	Zingiberaceae	3.9 g

Chronic restraint stress is one of the most recognized liver-*qi* stagnation models as it may mimic symptoms of depression in humans (Chen et al. 2008). Herein, we employed the chronic restraint stress model to investigate the effect of liver-*qi* stagnation on the metastasis of tumours in mice. Furthermore, based on the long-standing clinical applications of XYS for depression-associated conditions, we hypothesized that XYS may regulate stress hormones and inhibit chronic-stress-induced liver metastasis. Thus, in this study, we investigated the therapeutic effects of XYS using a xenograft mouse model of CRLM *in vivo*.

## Materials and methods

### Animals and cell line

A total of 40 four-week-old male BALB/C nu/nu mice (Shanghai SLAC Laboratory Animal Co. Ltd., Shanghai, China), weighing (18 ± 2 g), were used in the study. They were randomly divided into four groups (*n*= 10 per group): a blank-control (BC) group, a blank-stress (BS) group, an XYS-control (XC) group and an XYS-stress (XS) group. The animals were housed in specific-pathogen-free (SPF) conditions at room temperature (25 ± 1 °C) and relative humidity (50 ± 5%) with a 12 h light/dark cycle. All procedures were performed in accordance with and approved by the Animal Experimentation Ethics Committee of the Shanghai University of Traditional Chinese Medicine.

The human colon cancer cell line HT-29 was obtained from the Type Culture Collection of the Chinese Academy of Sciences (Shanghai, China). HT-29 cells were maintained in McCoy’s 5A medium (Gibco, Cat. no. 16600108, Grand Island, NY), supplemented with 10% foetal bovine serum (FBS) (Gibco, Cat. no. 16140071, Grand Island, NY), 100 U/mL penicillin and 100 μg/mL streptomycin (Gibco, Cat. no. 15070063, Grand Island, NY) at 37 °C in a humidified atmosphere of 5% CO_2_ and 95% air.

### Preparation of the XYS decoction

XYS granules were purchased from PuraPharm International Ltd (Hong Kong, Lot. HKP-08272, Cat. no. 14000592582). The compositional ingredients and crude medicinal components in XYS are shown in Table 1. The XYS granules were prepared according to the good manufacturing practice (GMP) guidelines of the Ministry of Food and Drug Safety (MFDS). The dose of XYS used in the current study was decided based on clinical use. In general, the dosage for a 60 kg human adult is 10 g XYS granules per day according to the instructions of manufacturer. Considering the conversion factor between humans and mice, we used a dosage of 1516.67 mg/kg bodyweight per day in the mice used in this study (dose for mice = 9.1 × clinical dose for humans (10 g XYS granules for 60 kg human)=1516.67 mg/kg) (Xu et al. 2002). For administration, XYS granules were dissolved in distilled water.

### *In vivo* metastasis model

Mice were injected with HT-29 colon cancer cells in the spleen to produce liver metastasis. For this procedure, mice were anaesthetized with 2.5% pentobarbital sodium by peritoneal injection and placed in a supine position. After sterilization of the surgical area, an abdominal incision paralleling the left subcostal margin was made. Tumour cells (1 × 10^6^) in 100 μL phosphate-buffered saline (PBS) solution were injected into the spleen using a 29 G needle. The spleen was then returned into the peritoneal cavity, and the wound was closed with sutures.

### Chronic restraint stress procedure and XYS treatment

The mice in the stress groups were exposed to immobilization stress, and were restrained in a plastic cylinder (height: 12 cm; diameter: 3 cm) for 2 h per day for 28 days commencing seven days before tumour cell injection. Mice were restrained in a confined space that prevented them from moving freely, but the restraints did not press on them or cause pain or wounding. The XYS decoction was administered to mice in the XC and XS group via gavage (30 mg/0.2 mL) 30 min before chronic immobilization stress for 28 days commencing seven days before tumour cell injection. The mice in the BC and BS groups were given distilled water.

### Flow cytometry analysis of spleen tumour tissue

Tissue from the spleen tumour was minced and washed repeatedly with PBS. The resulting slurry was subjected to partial enzyme digestion (DNAse, Roche, Cat. no. 10104159001, Basel, Switzerland; Pronase, Roche, Cat. no. 10165921001, Basel, Switzerland; Collagenase, Gibco BRL, Cat. no. 17100, Schwalbach, Germany), passed through a 100 μm nylon mesh, and spun on a density gradient (Ficoll-Paque, Amersham Biosciences, Cat. no. 17544652, Piscataway, NJ). Cells at the interface were collected and washed twice in PBS. The resulting single-cell suspensions were incubated with fluorescein isothiocyanate (FITC)-conjugated anti-mouse/human CD11b (BioLegend, Cat. no. 101206, San Diego, CA), phycoerythrin (PE)-conjugated anti-mouse F4/80 (BioLegend, Cat. no. 123110, San Diego, CA) and Mouse MDSC Flow Cocktail 2 with Isotype Ctrl (BioLegend, Cat. no. 147003, San Diego, CA). Antibodies were diluted 1:100 in flow cytometric buffer (PBS with 5% FBS) and incubated with cells for 1 h at 4 °C. Cells were then washed and resuspended in antibody-free flow cytometric buffer. Flow cytometric measurement of fluorescence emission was performed on a total of 10,000 cells per dissociated tumour tissue by a Beckman/Coulter Epics XL-MCL flow cytometer with EXPO32 software (Beckman, CA).

### Gene expression analysis using real-time polymerase chain reaction (PCR)

Quantitative real-time reverse transcription-PCR (RT-PCR) was used to quantify interleukin-6 (IL-6), vascular endothelial growth factor (VEGF), matrix metalloproteinase-9 (MMP-9) and transforming growth factor β (TGF-β) mRNA isolated from tumour tissue from the spleen. Total RNA was extracted using TRIzol reagent (Invitrogen, Cat. no. 15596026, Carlsbad, CA), and used for cDNA synthesis with 1 μg RNA and SYBR^®^ Premix Ex Taq™ II (Takara, Cat. no. RR420, Shiga, Japan). Samples were assayed in triplicate. Differences in gene expression were calculated using the comparative *C*_T_ method (2^−ΔΔ^*^CT^*) (Livak and Schmittgen 2001). The primers (Generay, Shanghai, China) used in our study are shown in Table 2.

**Table 2.** Sequences of the primers used in the real-time PCR analysis.

Gene	Primer sequence (5′–3′)
IL-6	F: TAGTCCTTCCTACCCCAATTTCC
	R: TTGGTCCTTAGCCACTCCTTC
VEGF	F: GCCAGACAGGGTTGCCATAC
	R: GGAGTGGGATGGATGATGTCAG
MMP-9	F: CTGGACAGCCAGACACTAAAG
	R: CTCGCGGCAAGTCTTCAGAG
TGF-β	F: AGACCACATCAGCATTGAGTG
	R: GGTGGCAACGAATGTAGCTGT
GAPDH	F: AGGTCGGTGTGAACGGATTTG
	R: TGTAGACCATGTAGTTGAGGTCA

### Western blot

The western blot analysis was conducted with a GAPDH monoclonal antibody (1:4000, Abcam, ab181602, Shanghai, China) used as an internal control. The primary antibodies used were the TGF-β antibody (1:1000, Abcam, ab189778, Shanghai, China), IL-6 antibody (1:1000, Abcam, ab208113, Shanghai, China), VEGF (1: 1000, Abcam, ab1316, Shanghai, China) and MMP-9 (1:1000, Abcam, ab38898, Shanghai, China), respectively.

### Immunohistochemistry

Antibody staining was performed on histological sections of formalin-fixed liver metastases. Tumour sections were incubated overnight at 4 °C with the following primary antibodies: anti-VEGF (1:100, Abcam, ab1316, Shanghai, China) and anti-CD31 (1:100, Abcam, ab28364, Shanghai, China). The slides were rinsed and incubated in biotinylated secondary antibody (1:500, Abcam, ab6720 or ab6788, Shanghai, China). The intensity of staining for VEGF and CD34 expression was measured by Image-J with IHC profiler (Varghese et al. 2014).

### Statistical analysis

Results were reported as the mean ± SEM. The data were analysed by one-way ANOVA test followed by Tukey's *post hoc* test. A *p* value <0.05 was considered statistically significant.

## Results

### Effects of chronic restraint stress and XYS on liver metastasis

Mice were injected with HT-29 colon cancer cells into the spleen to facilitate liver metastasis. To delineate the role of chronic restraint stress in the progression of liver metastasis, mice in the BS and XS groups were exposed to chronic restraint stress for 2 h per day for 28 days commencing seven days before tumour cell injection. In addition, we examined the inhibitory effect of XYS on the growth of tumours in a xenograft mouse model of CRLM. XYS was given orally each day for 28 days commencing seven days before tumour cell injection. After 28 days, mice were sacrificed to evaluate tumour growth.

**Figure 1.**
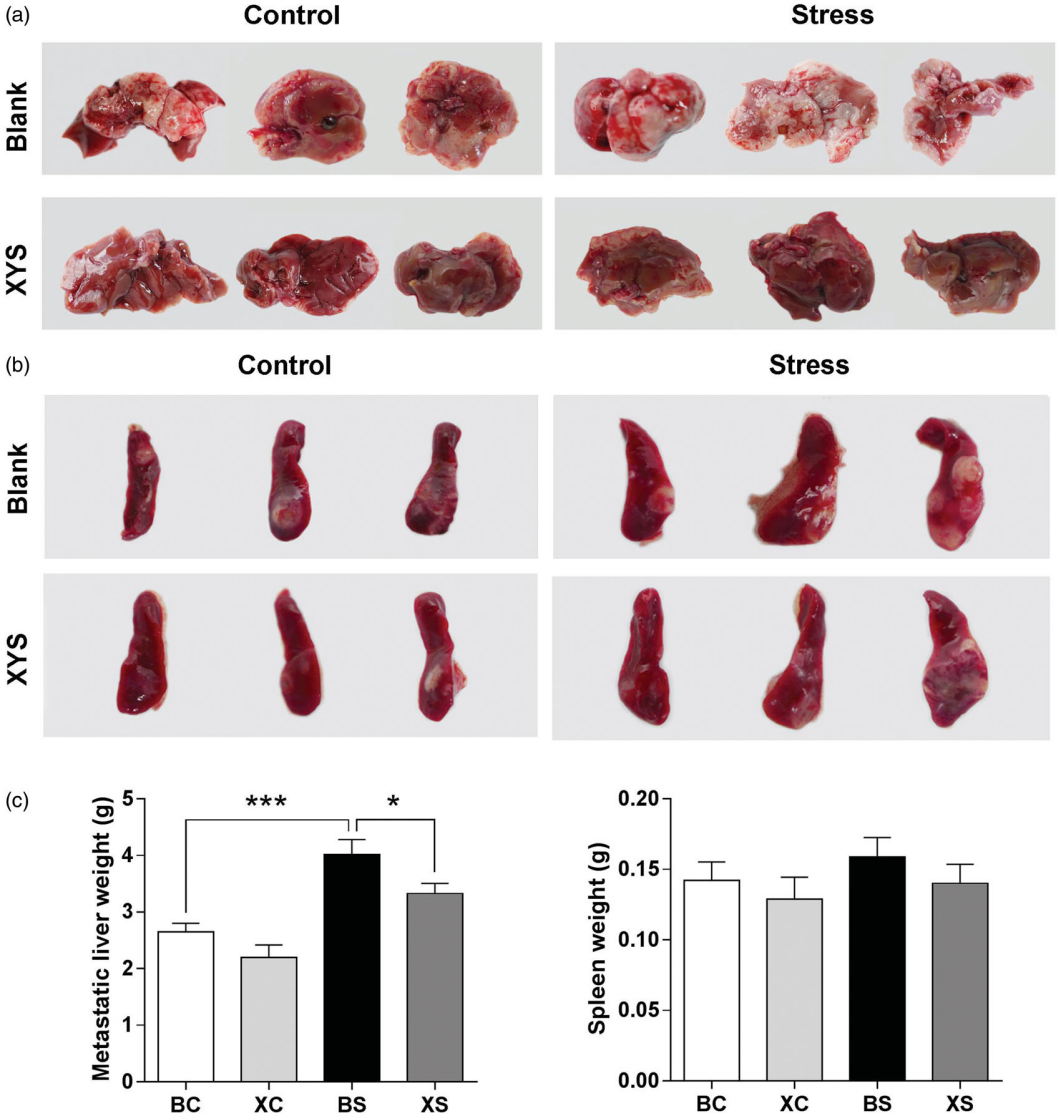
Effect of chronic stress and XYS on liver metastasis in a CRC model. (a) Representative images of the metastatic liver taken on day 28 after tumour cell injection. (b) Representative images of the spleen taken on day 28 after tumour cell injection. (c) Data represent average liver (left) and spleen (right) weights for the four groups. *p< 0.05, ***p< 0.001. BC: blank-control; BS: blank-stress; XC: XYS-control; XS: XYS-stress.

The spleen weight between the four groups did not differ significantly (*p*> 0.05; Figure 1). However, a significant increase in metastatic liver weight was observed in BS mice (1.5-fold change: 4.01 ± 0.27 g in BS group vs. 2.65 ± 0.15 g in BC group; Figure 1). XYS treatment appeared to have an inhibitory effect on this process and attenuated the increase of liver metastasis induced by chronic restraint stress. Compared to mice in the BS group, mice in the XS group had fewer liver metastases. In fact, the metastatic liver weight of mice in the XS group was significantly lower than that of mice in the BS group (XS vs. BS: 3.33 ± 0.18 g vs. 4.01 ± 0.27 g). We observed no significant difference in liver metastasis between the XC group and BC group (*p*> 0.05).

### Effects of chronic restraint stress and XYS on the tumour microenvironment

**Figure 2.**
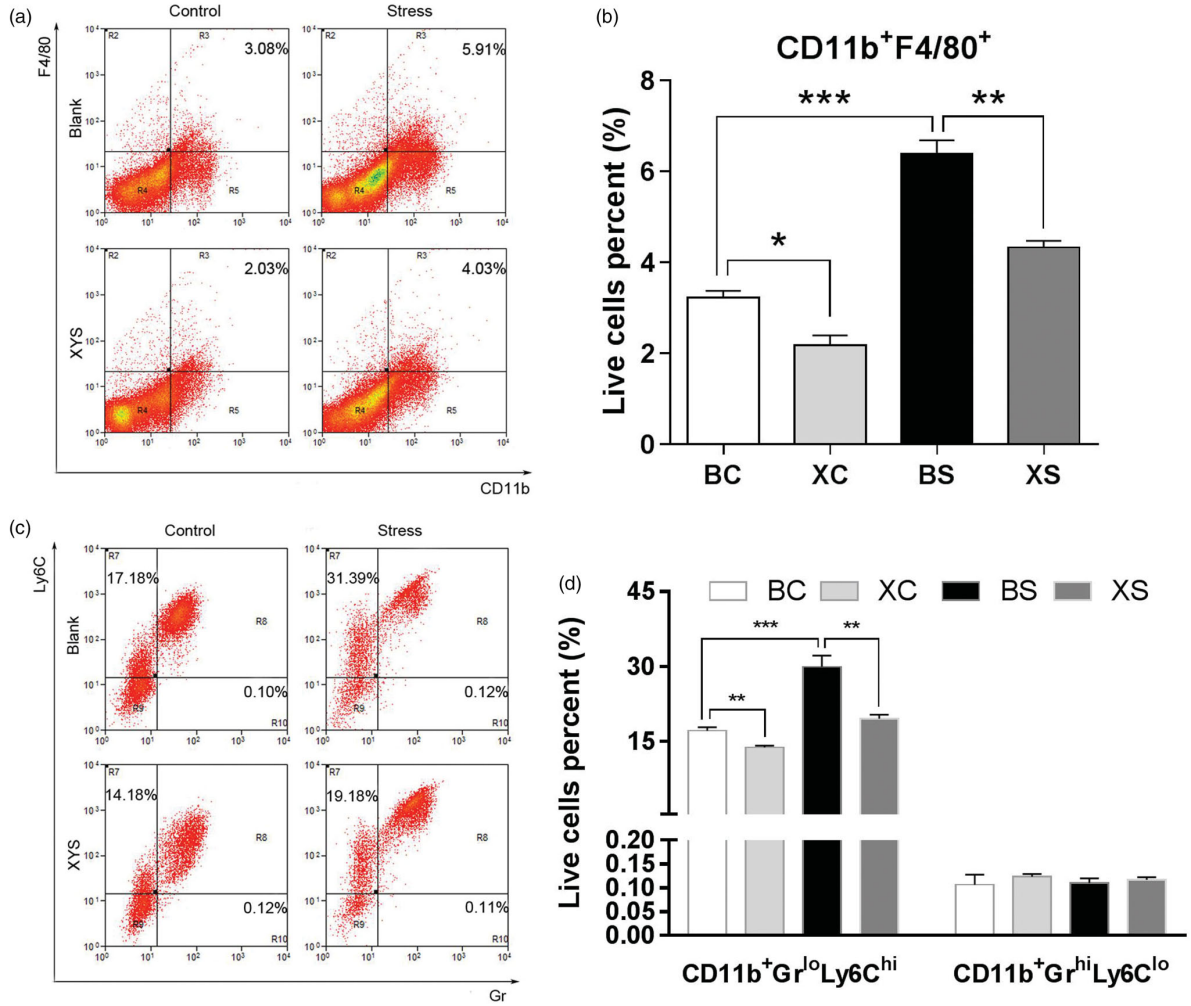
Effects of chronic restraint stress and XYS on the tumour microenvironment. (a) CD11b^+^F4/80^+^ cells were quantified by flow cytometry in disaggregated HT-29 primary spleen tumour harvested 28 days after cell injection. (b) Data represent the average CD11b^+^F4/80^+^ composition. (c) CD11b^+^Gr^lo^Ly6C^hi^ and CD11b^+^Gr^hi^Ly6C^lo^ cells were quantified by flow cytometry in disaggregated HT-29 primary spleen tumour harvested 28 days after cell injection. (d) Data represent the average CD11b^+^Gr^lo^Ly6C^hi^ composition. *p< 0.05, **p< 0.01 and ***p< 0.001. BC: blank-control; BS: blank-stress; XC: XYS-control; XS: XYS-stress.

To investigate whether chronic restraint stress and XYS affect the tumour microenvironment, flow cytometry was employed to quantify the cell composition of primary tumours harvested from the spleen. As shown in Figure 2, chronic restraint stress significantly increased CD11b^+^F4/80^+^ cell infiltration (1.98-fold change: 6.39 ± 0.29% of live cells in the BS group vs. 3.23 ± 0.15% of live cells in the BC group; *p*< 0.001). Chronic restraint stress also increased the CD11b^+^Gr^lo^Ly6C^hi^ cell infiltration (BS vs. BC: 29.96 ± 1.29% of live cells vs. 17.15 ± 0.38% of live cells; *p*< 0.001). In order to observe the effect of XYS on the tumour microenvironment, we analysed the cell composition of spleen tumour tissue harvested from mice treated with XYS. XYS treatment significantly decreased the CD11b^+^F4/80^+^ macrophage population in the primary tumour tissue (XS vs. BS: 4.33 ± 0.15% of live cells vs. 6.40 ± 0.29% of live cells; *p*= 0.001; XC vs. BC: 2.19 ± 0.21% of live cells vs. 3.23 ± 0.15% of live cells; *p*= 0.032). Also, a significant reduction in CD11b^+^Gr^lo^Ly6C^hi^ cells was noted following XYS treatment (XS vs. BS: 19.55 ± 0.46% of live cells vs. 29.96 ± 1.29% of live cells; *p*< 0.001; XC vs. BC: 13.81 ± 0.19% of live cells vs. 17.15 ± 0.38% of live cells; *p*= 0.044). However, chronic restraint stress-induced recruitment of myeloid-derived suppressor cells (MDSCs) was specific to Gr^lo^Ly6C^hi^, as no significant increase was observed for other types, such as Gr^hi^Ly6C^lo^ polymorphonuclear neutrophil-like suppressor cells.

### Molecular mechanism of chronic restraint stress and XYS on liver metastasis

**Figure 3.**
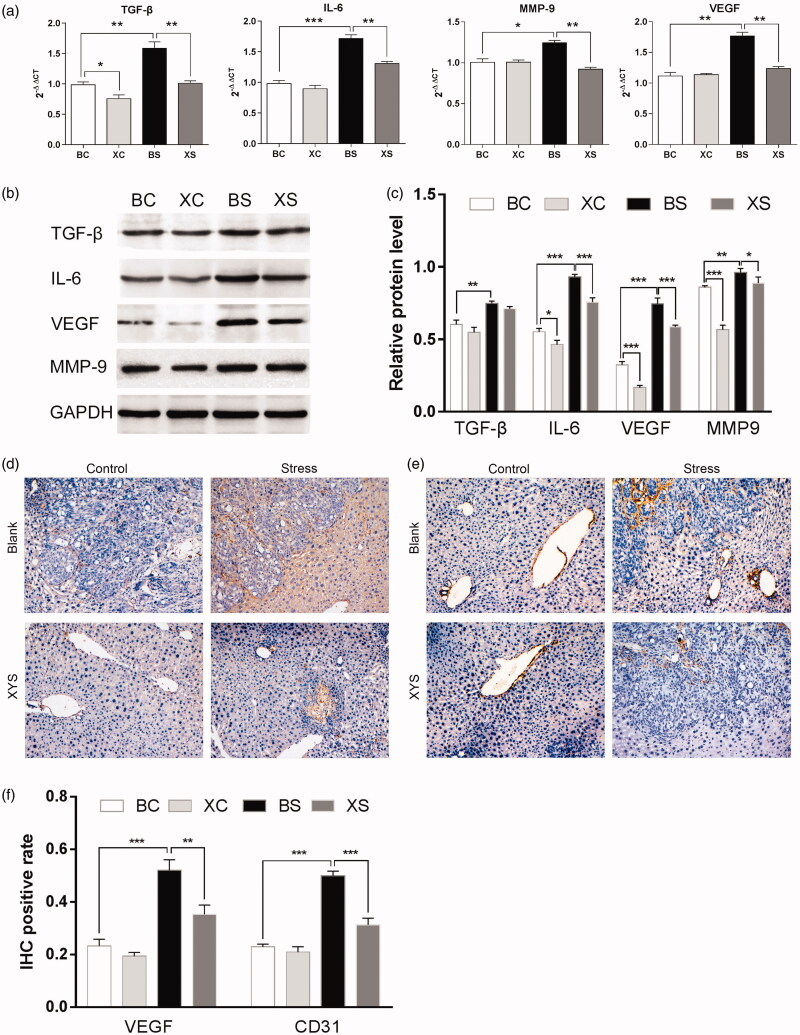
Effects of chronic restraint stress and XYS on liver metastasis in a CRC model. (a) mRNA expression of TGF-β, IL-6, MMP-9 and VEGF in spleen tumours relative to mice in the BC group. (b) Protein expression of TGF-β, IL-6, MMP-9 and VEGF in spleen tumours. Glyceraldehyde 3-phosphate dehydrogenase (GAPDH) was used as a loading control. (c) Relative protein expression levels. GAPDH was used as an internal standard. (d) Immunohistochemical detection of VEGF protein expression in metastatic liver tumour tissue. (e) Immunohistochemical detection of CD31 protein expression in metastatic liver tumour tissue (magnification ×200). (f) IHC positive rate of VEGF and CD31 in metastatic liver tumours (magnification ×200). *p< 0.05, **p< 0.01 and ***p< 0.001. BC: blank-control; BS: blank-stress; XC: XYS-control; XS: XYS-stress.

To determine the effects of chronic restraint stress and XYS on liver metastasis, we examined the expressions of TGF-β, IL-6, MMP-9 and VEGF in spleen tumours by RT-PCR and Western blot analysis. We found that TGF-β, IL-6, MMP-9 and VEGF mRNA expressions significantly increased in mice in the BS group as compared to mice in the BC group. Treatment of mice undergoing chronic restraint stress with XYS significantly reduced TGF-β, IL-6, MMP-9 and VEGF mRNA expressions compared to mice in the BS group (Figure 3(a)).

In addition, we observed increased protein expressions of TGF-β, IL-6, MMP-9 and VEGF in mice exposed to chronic restraint stress (BS vs. BC), which was ameliorated by treatment with XYS (XS vs. BS) (Figure 3(b,c)).

Previous research has suggested that chronic restraint stress promotes angiogenesis, thus promoting tumour metastasis (Feng et al. 2012; Ji et al. 2019). Hence, we examined the protein expressions of VEGF and CD31 in hepatic metastatic tissue. Treatment of chronic restraint stress mice with XYS significantly reduced VEGF and CD31 protein expressions (Figure 3(d–f)). Collectively, our findings suggest that XYS treatment can weaken the effect of chronic restraint stress on the promotion of cancer cell invasion and metastasis.

## Discussion

Despite advanced diagnostic and therapeutic methods, liver metastasis still poses a significant challenge in prolonging patient survival in CRC. The cumulated incidence for liver metastasis is 15% at 5 years and 17% at 10 years (Landreau et al. 2015). Hence, multi-modal therapies are essential for effective treatment. Recently, psychological stress, such as depression, has been emphasized as a cause of tumour growth, invasion and metastasis (Yi and Syrjala 2017).

Psychological stress has also been emphasized as a main cause of disease in the TCM system. XYS is a popular TCM preparation used clinically for the treatment of mental illness in China. In fact, the name ‘Xiaoyaosan’ translates as ‘free and unfettered’ (Li et al. 2017; Yan et al. 2018).

In the present study, a mouse model of liver metastasis from CRC was established to evaluate the effect of chronic restraint stress and the therapeutic actions of XYS *in vivo*. The results clearly showed that XYS has an inhibitory effect on the development of liver metastasis in mice exposed to chronic restraint stress. In our study, the mean weight of the metastatic liver was significantly increased in the stress groups compared to the control groups. However, treatment with XYS resulted in a decreased metastatic liver weight compared to the BS group.

The role of myeloid cells in the metastatic dissemination of colon cancer cells to the liver has been extensive studied previously (Kitamura et al. 2010; Hirai et al. 2014), and research has established that this process is associated with tumour-associated macrophages (TAMs) (Zhou et al. 2010) and MDSCs (Jin et al. 2013). TAMs play an important role in the progression of inflammatory conditions to cancer, and they are capable of infiltrating the tumour microenvironment (Wu et al. 2018). Also, TAMs have been shown to promote the expansion of colon cancer cells in the liver (Kitamura et al. 2010). Meanwhile, MDSCs, which are a type of inhibitory immune cell, have been reported to accumulate during tumour progression, traumatic stress and psychological stress (Ostrand-Rosenberg and Sinha 2009; Giakoustidis et al. 2015; Zhou et al. 2018). MDSCs in mice comprise a heterogeneous population of immature myeloid cells (CD11b^hi^Gr-1^+^), and can be further classified into monocytic (Ly6C^high^Ly6G^low^) and granulocytic (Ly6C^low^Ly6G^high^) subsets (Youn et al. 2008). In most tumour models, the expansion of MDSCs predominately involves the granulocytic subset (70–80%). Both subsets inactivate the immune response via several pathways, including l-arginine depletion through arginase-1 and inducible nitric oxide synthase activity, increased generation of reactive oxygen species, and the production of immunosuppressive cytokines, such as TGF-β (Talmadge 2007; Trikha and Carson 2014; Tcyganov et al. 2018). Accordingly, we hypothesized that chronic restraint stress may have an impact on myeloid cells. Thus, as we expected, flow cytometry demonstrated that chronic restraint stress increased TAM and MDSC levels with increasing liver metastasis. Furthermore, in accordance with most published reports, the chronic restraint stress-induced recruitment of MDSCs was specific to the granulocytic subset, as no significant increase was observed for the Gr^high^Ly6C^low^ subset. The current study confirms previous indications that chronic stress can influence cancer metastasis and drive changes in TAM and MDSC recruitment, thereby altering gene expression within the primary tumour (Hassan et al. 2013; Nan et al. 2018; Myers et al. 2019). In addition, our findings demonstrate that administration of XYS may significantly reduce the recruitment of TAMs and MDSCs. These findings suggest that XYS may inhibit liver metastasis through the regulation of myeloid cell recruitment.

The inhibitory effect of XYS was accompanied by downregulated expression of TGF-β, IL-6, MMP-9 and VEGF in spleen tumours, as well as suppression of VEGF and CD31 proteins in hepatic metastatic tissue. These results suggest that the inhibition of angiogenesis is involved in the inhibitory effect of XYS on liver metastasis in mice exposed to chronic restraint stress. In summary, XYS may decrease the recruitment of TAMs and MDSCs, as well as the expression of metastasis-related genes.

## Conclusions

This study reports for the first time that XYS may successfully inhibit chronic-stress-induced liver metastasis. In this regard, our findings provide new information that may lead to the design of novel treatments for CRC metastasized to the liver, and that these treatments may be particularly relevant for patients suffering with depression. However, the exact herbal components in XYS that inhibit cancer metastasis were not elucidated and this is a limitation of the present study. Furthermore, how XYS affects the immunological response and the related molecular mechanisms require further research.
